# Platinum accumulation in chemotherapy: toxicity mechanisms, challenges, and mitigation strategies

**DOI:** 10.1007/s10534-026-00812-y

**Published:** 2026-04-29

**Authors:** Yier Lai, Guodong Qiu, Zhiwei Zheng, Xiaoting Huang, Pi Guo, Yuling Zhang, Ling Fang

**Affiliations:** 1 Pharmacy Department, Cancer Hospital of Shantou University Medical College, Shantou, 515041 Guangdong China; 2 Shantou University Medical College, Shantou, 515041 Guangdong China; 3 Pharmacy Intravenous Admixture Service, Cancer Hospital of Shantou University Medical College, Shantou, 515041 Guangdong China; 4 Department of Preventive Medicine, Shantou University Medical College, Shantou, 515041 Guangdong China; 5 Research Institute of Clinical Pharmacy, Shantou University Medical College, Shantou, 515041 Guangdong China

**Keywords:** Platinum accumulation toxicity, Mitochondrial dysfunction, Oxidative stress, Drug resistance

## Abstract

Platinum-based chemotherapeutic agents constitute the cornerstone of treatment for a wide range of solid tumors. However, residual platinum can persist in the body long after treatment cessation, leading to progressive accumulation, chronic multi-system toxicity, and significant impairment of patients' quality of life. The insidious onset and lack of specific monitoring methods often lead to the oversight of this treatment-related metal accumulation toxicity. This article reviews the environmental and occupational exposure risks of platinum, its unique pharmacokinetic properties, and elucidates how its irreversible binding to proteins and accumulation within mitochondria form the chemical basis for its long-term toxicity. It further elaborates on the core mechanisms underlying platinum accumulation-mediated multi-organ toxicity, including nephrotoxicity, neurotoxicity, and ototoxicity. The aim is to provide insights and directions for improving the long-term prognosis of cancer survivors by clarifying the toxicological mechanisms, clinical impacts, and future strategies for mitigating platinum-induced metal accumulation.

## Introduction

Heavy metals are defined as elements with a density exceeding 4.5 g/cm^3^ and high atomic weight (Bharti and Sharma 2022). They are generally classified into two categories: the first comprises essential trace elements such as iron (Fe), manganese (Mn), zinc (Zn), and copper (Cu), which are indispensable for maintaining normal physiological functions and homeostasis (Zoroddu et al. 2019). The second category includes non-essential elements lacking clearly defined physiological functions in humans, such as cadmium (Cd), lead (Pb), mercury (Hg), and platinum (Pt), as well as metalloids like arsenic (As), which is commonly included in the research system of heavy metal toxicity (Abd Elnabi et al. 2023).

Certain non-essential heavy metals (e.g., Pb, Cd, As, Hg) exhibit strong cumulative properties, long biological half-life, and water solubility, exerting well-documented toxicity to humans and ecosystems even at minimal environmental exposure levels (Budi et al. 2024). Their core toxic mechanisms include covalent binding to DNA and proteins, disruption of cellular metabolic processes, induction of DNA damage, protein inactivation, oxidative stress, and mitochondrial dysfunction. For instance, Cd specifically accumulates in the kidneys, impairing renal tubular reabsorption function, eventually progressing to chronic kidney disease; As inhibits enzyme activity via binding to protein thiol groups, blocks DNA, and induces chromosomal aberrations; Pb suppresses heme synthesis by inhibiting δ-aminolevulinic acid dehydratase (ALAD), leading to iron deficiency anemia (Kotnala et al. 2025; Tchounwou et al. 2012). Additional toxic mechanisms include ionic mimicry and structural disruption, such as Pb interfering with neuronal signal transduction via calcium substitution, and Hg causing irreversible inactivation of key metabolic enzymes through binding to thiol groups (Jaishankar et al. 2014).

Platinum Group Metals (PGMs) are released into the environment via abrasion of automotive catalytic converters, leading to rising concentrations in soil and aquatic systems. Exposure to PGMs has been shown to significantly inhibit plant growth and induce DNA damage in the wetland plant Sphagnum, and trigger pathological changes including adrenal vacuolization, renal glomerular atrophy, and splenic white pulp expansion in the organs of rat models (Gagnon et al. 2006).

Among PGMs, platinum is used across the automotive industry, chemical catalysis, clinical medicine, and dental restoration. Accordingly, the health risks of human platinum exposure and accumulation have become a key research focus in toxicology and oncology pharmacology.

Elemental platinum (Pt⁰) exhibits extremely high geochemical stability in natural environments (e.g., unpolluted soil, fresh water) lacking strong complexing ligands (e.g., Cl⁻, S_2_O_3_^2^⁻). However, Pt⁰-bearing nano- and micro-particles, predominantly emitted from automotive three-way catalytic converter abrasion and fossil fuel combustion, continuously accumulate in soils and sediments of industrial zones and along roadways. Once these particles enter ligand-rich media (e.g., chloride-bearing surface water, organic-rich pore water in sediments), they undergo oxidative dissolution and transform into bioavailable Pt(II)/Pt(IV) complexes. These complexes are resistant to metabolic degradation in living organisms, allowing them to bioaccumulate and be transferred along the food chain (Reith et al. 2014; Colombo et al. 2008; Rauch and Morrison 2000). For example, Pt can accumulate to concentrations exceeding 38 ng/g dry weight in the freshwater isopod *Asellus aquaticus* exposed to traffic-contaminated sediments (Moldovan et al. 2001), with the accumulated Pt further transferred to top predators such as wild raptors via predation: studies have detected 0.2 ng/g Pt in raptor liver and kidney tissues, and a maximum concentration of 3.4 ng/g in their blood samples (Ek et al. 2004). In humans, diet is the primary route of non-occupational Pt exposure, with an average dietary Pt intake of 1.44 μg per day estimated for adults in Sydney, Australia (Vaughan and Florence 1992). Absorbed Pt can bind to metallothionein (MT) in renal tissues, leading long-term retention in the kidneys. While high-dose exposure to Pt-based chemotherapeutic agents has well-documented nephrotoxicity, no conclusive causal link has been established to date between chronic low-level environmental Pt exposure and adverse renal outcomes in humans (Rauch and Morrison 2000; Reith et al. 2014; Herr et al. 2003).

Monitoring data from occupational exposure cohorts further confirm the long-term accumulation of platinum in the human body. Urinary platinum concentration measurements in 34 workers from the platinum industry revealed that the median urinary platinum excretion in exposed workers was 1000-fold higher than that in unexposed controls. Urinary platinum excretion follows a biphasic half-life: the first phase has a half-life of approximately 50 h, while the clearance of the long-term retention fraction occurs over approximately 24 days. Notably, in workers with high exposure, urinary platinum levels remained 25-fold higher than those in the control group even 2–6 years after cessation of exposure (Schierl et al. 1998). This long-term accumulation property is associated with a wide spectrum of adverse health outcomes, including hypersensitivity reactions, asthma, alopecia, miscarriage, and dermatitis (Ravindra et al. 2004; Reith et al. 2014). Beyond environmental and occupational exposure, iatrogenic platinum exposure in daily life is equally non-negligible. Analysis of urinary platinum concentrations in 84 patients with skin diseases identified noble metal dental alloy restorations (containing gold, platinum, and palladium) as the most critical determinant of human platinum exposure. The number of restorations was significantly and positively correlated with urinary platinum concentration, with each additional restoration associated with an approximate 35% increase in urinary platinum levels (Herr et al. 2003). Furthermore, urinary platinum excretion remained sevenfold higher than pre-implantation levels 3 months after restoration placement, whereas no significant increase in urinary excretion of palladium or gold was observed post-implantation (Begerow 1999). These findings demonstrate that non-therapeutic platinum exposure, whether from environmental, occupational, or daily life settings, results in platinum entry into the human body and subsequent long-term systemic accumulation.

Unlike exposure to other environmental heavy metals (e.g., Pb, Hg, Cd) and passive platinum exposure, systemic chemotherapy with clinical platinum-based antineoplastic agents represents the dominant source of active human platinum exposure, as well as the core pathway leading to systemic platinum accumulation in the human body.

Platinum-based chemotherapeutic agents, including first-generation cisplatin, second-generation carboplatin and nedaplatin, third-generation oxaliplatin and lobaplatin, as well as novel platinum agents (e.g., ormaplatin, satraplatin), exert their antineoplastic effects primarily by targeting nuclear DNA. The formation of platinum–DNA adducts mediates tumor cell necrosis or apoptosis, establishing these agents as the cornerstone of treatment for a wide range of solid tumors, including testicular cancer, ovarian cancer, lung cancer, and colorectal cancer (Johnstone et al. 2016).

Despite their well-established antineoplastic efficacy, platinum-based agents have poor tumor selectivity, frequently causing acute adverse reactions such as acute kidney injury, myelosuppression, and gastrointestinal toxicities. These toxicities often necessitate dose reduction or premature discontinuation of chemotherapy regimens.

**Fig. 1:**
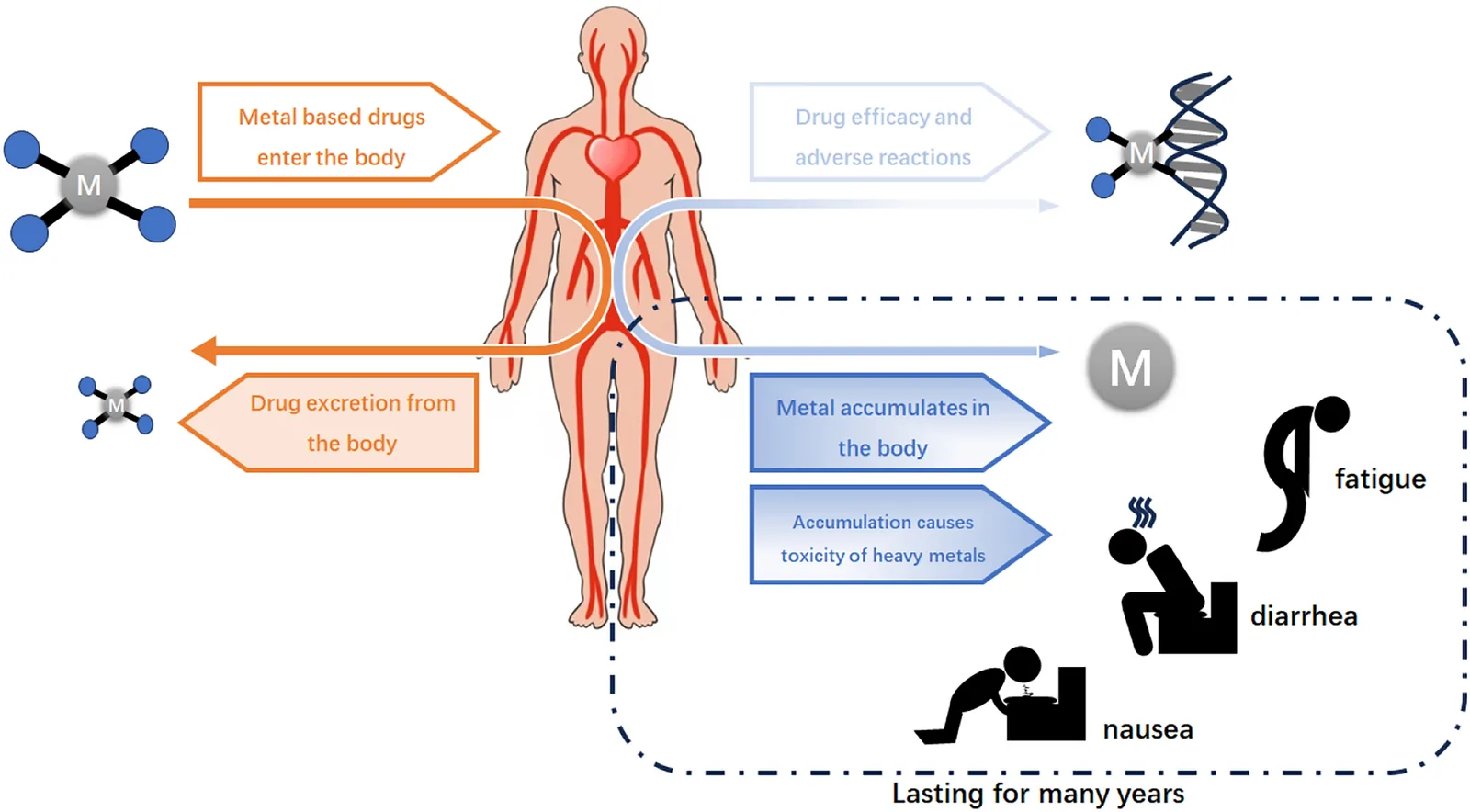
Toxic effects caused by the accumulation of metal-based drugs

More importantly, the residual platinum after chemotherapy will accumulate in the body over a long period of time—Numerous clinical studies have confirmed that even after the theoretical complete elimination cycle (5 elimination half-lives) of platinum-based agents, platinum concentrations significantly above the healthy baseline remain detectable in patients (Zhang et al. 2020). A large-scale 20-year cohort study of 458 cisplatin-treated testicular cancer survivors further demonstrated that serum platinum levels remained significantly elevated compared with surgery-only, chemotherapy-naive controls even 20 years post-chemotherapy, with a clear dose-dependent positive correlation between residual platinum levels and cumulative cisplatin dose (Hjelle et al. 2015). Brouwers et al. enrolled 45 patients with solid tumors 8–75 months after the completion of platinum-based chemotherapy, and the test results showed that the plasma platinum concentration of patients was significantly higher than that of healthy controls with no platinum exposure history. Of the total plasma platinum, 24% was present as an ultrafilterable, biologically active free form, which retained binding activity toward biomacromolecules (Brouwers et al. 2008). The long-term accumulation of platinum may be associated with a series of delayed, irreversible long-term health risks (Fig. 1).

To date, extensive research has focused on the acute toxicities, single-organ injury mechanisms, and chemoresistance of platinum-based agents. However, there remains a lack of systematic collation and integration of the clinical characteristics of long-term platinum accumulation and its underlying mechanisms of long-term toxicity. Meanwhile, clinical practice predominantly focuses on acute adverse reactions during chemotherapy, with insufficient attention paid to the long-term accumulation risks of residual platinum after drug elimination. On this basis, this review systematically summarizes the pharmacokinetic evidence and biological basis of long-term in vivo accumulation of platinum-based chemotherapeutic agents, elaborates on the mechanisms of organ toxicity mediated by platinum accumulation, clarifies the core association between insufficient platinum accumulation in tumor cells and chemoresistance, and finally concludes the key challenges and future research directions in this field, aiming to provide a theoretical basis for individualized adjustment of clinical chemotherapy regimens, early screening and intervention of long-term toxicity caused by platinum accumulation, and long-term health management of cancer survivors.

## Pharmacokinetics of platinum accumulation

The in vivo pharmacokinetic characteristics of platinum-based agents may determine their tissue accumulation levels, anti-tumor efficacy, and related toxicity. After intravenous administration, the agents enter the systemic circulation and rapidly undergo covalent binding to plasma biomacromolecules within a short period, with human serum albumin (HSA) as the primary binding target.

### Plasma protein binding properties and accumulation potential

Following intravenous injection into the systemic circulation, platinum-based agents form irreversible covalent bonds with plasma biomacromolecules within minutes to hours, predominantly with HSA. The plasma protein binding (PPB) is related to the in vivo distribution profile, clearance efficiency, and long-term accumulation risk of the agents. Significant inter-agent differences exist in the protein binding characteristics and tissue accumulation capacity among different generations of platinum-based agents.

Protein-bound complexes formed by cisplatin can be retained in the body for a long time with a significantly prolonged elimination half-life, which serves as the core material basis for its long-term cumulative toxicities including nephrotoxicity and ototoxicity. Oxaliplatin binds to both erythrocytes and serum albumin: platinum complexes formed via erythrocyte binding have a half-life of up to 12–50 days, while agents bound to serum albumin can form a “long-term reservoir” in the body, continuously releasing free platinum to target tissues such as peripheral nerves, and ultimately mediating chronic cumulative neurotoxicity. Carboplatin has stronger water solubility due to the hydrophilic cyclobutanedicarboxylic acid ligand in its molecular structure, and is mainly excreted rapidly in prototype form via the kidneys. Its PPB is significantly lower than that of cisplatin, resulting in a lower risk of cumulative toxicity (Graham et al. 2000; O'Dwyer et al. 2000; Esteban-Fernández et al. 2008). The second-generation nedaplatin and third-generation lobaplatin have lower PPB and faster in vivo clearance, with lower risks of related cumulative toxicity.

### Regulatory effects of transporters on drug accumulation and tissue distribution

Transmembrane transporters are the core molecules regulating the cellular uptake and efflux of platinum-based agents, which influence the accumulation levels of the agents in tumor cells and normal target organs, thereby affecting chemotherapy efficacy and toxicity risk. According to their functions, transporters can be divided into two major categories: uptake transporters and efflux transporters.

Among uptake transporters, Copper Transporter 1 (CTR1) is the primary transporter mediating the uptake of platinum-based agents by tumor cells. Its cell membrane expression level is significantly positively correlated with intracellular platinum accumulation and chemotherapy sensitivity of tumor cells. For example, downregulated CTR1 expression has been confirmed in cisplatin-resistant ovarian cancer cell lines, which directly leads to reduced drug uptake and insufficient platinum accumulation in tumor cells, ultimately triggering chemotherapy resistance (Kalayda et al. 2012). Organic Cation Transporter 2 (OCT2) is highly expressed in renal proximal tubular epithelial cells and the basilar membrane of cochlear hair cells. It is the key molecule mediating the specific uptake and accumulation of cisplatin in renal and cochlear tissues, as well as the core initiating factor of cisplatin-induced acute kidney injury and ototoxicity (Harrach and Ciarimboli 2015). Among efflux transporters, Copper Transporting ATPase α (ATP7A) and Copper Transporting ATPase β (ATP7B) are the core molecules regulating the cellular efflux of platinum-based agents. Upregulated expression of these two transporters can significantly accelerate the efflux of platinum agents from cells and reduce intracellular platinum accumulation, which not only impairs anti-tumor efficacy and mediates chemotherapy resistance, but also affects cumulative toxicity by altering the tissue distribution profile of the agents. Clinical studies have confirmed that among ovarian cancer patients, the objective response rate to platinum-based chemotherapy in patients with ATP7B-positive tumors is significantly lower than that in patients with ATP7B-negative tumors. Moreover, the median overall survival of ATP7B-positive patients is only 33 months, which is significantly shorter than the 66 months of ATP7B-negative patients (Hall et al. 2008).

### Intracellular metabolic activation and excretion characteristics

After entering cells, platinum-based agents rapidly undergo hydrolytic activation in the low-chloride intracellular environment, generating positively charged active hydrated platinum ions (Pt^2^⁺). On the one hand, active platinum ions bind to the genomic DNA of tumor cells to form platinum–DNA adducts, block DNA replication and transcription, and ultimately mediate tumor cell apoptosis/necrosis to exert anti-tumor effects. On the other hand, as a typical soft acid, platinum ions have an extremely high affinity for biomacromolecules containing soft base atoms such as sulfur (S) and nitrogen (N), and can form irreversible covalent bonds with sites including the cysteine thiol group (-SH), histidine imidazole group, and methionine thioether group (-S-CH_3_) of proteins in normal cells (Corinti et al. 2020). Such platinum-biomacromolecule complexes have high chemical stability (Benedetti et al. 2011) and a long elimination half-life in vivo, which is the core molecular mechanism underlying the long-term accumulation and accumulation-related toxicity of platinum-based agents (Messori and Merlino 2016).

The kidney is the primary excretory organ for platinum-based agents and their metabolites, with the vast majority of the agents ultimately excreted via urine. Significant differences exist in renal excretion efficiency and elimination characteristics among different platinum-based agents. The core pharmacokinetic parameters of various platinum-based agents are summarized in Table 1 (Esteban-Fernández et al. 2008; Graham et al. 2000; O'Dwyer et al. 2000; McKeage 1995; Alassadi et al. 2022; Ivanova 2022).

**Table 1:** Pharmacokinetic characteristics of platinum-based drugs

Drug	Administration & Absorption	Distribution	Metabolism	Excretion
Cisplatin	Intravenous injection; negligible oral absorption	Rapid diffusion into tissues after IV infusion; Very high plasma protein binding (> 90%), resulting in low free drug concentration	Easily hydrolyzed in vivo to generate active metabolites, which bind to DNA for antitumor effect; not dependent on CYP450 enzyme system	Total Platinum: t₁/₂α = 13 min, t₁/₂β = 43 min, t₁/₂γ = 5.4 days Ultrafilterable Platinum: t₁/₂α = 6 min, t₁/₂β = 36 min Primarily renal excretion; Low excretion rate (23–50%); Renal impairment may affect its excretion
Carboplatin	Intravenous injection; negligible oral absorption	Low PPB (24–50%); High drug stability; Nephrotoxicity significantly lower than cisplatin	High metabolic stability, mainly exists as parent drug in vivo with few metabolites; not dependent on CYP450 enzyme system	Total Platinum: t₁/₂α = 22 min, t₁/₂β = 116 min, t₁/₂γ = 5.8 days Ultrafilterable Platinum: t₁/₂α = 23 min, t₁/₂β = 120 min Primarily excreted unchanged in urine; High excretion rate (54–82%), highly correlated with Glomerular Filtration Rate (GFR)
Nedaplatin	Intravenous injection; negligible oral absorption	Low PPB	Metabolized via non-enzymatic pathways	Total Platinum: t₁/₂α = 0.1–1 h; t₁/₂β = 2–13 h; Half-life of ultrafilterable platinum similar to total platinum. Renal excretion rate: 59.6%
Oxaliplatin	Intravenous injection; oral formulation has ~ 15% bioavailability (rarely used clinically)	Three-compartment distribution: protein-bound platinum (85% binding), ultrafilterable platinum, and red blood cell-bound platinum	Complex metabolism, generating multiple active metabolites; not dependent on CYP450 enzyme system	Ultrafilterable Platinum: t₁/₂ = 24.2 ± 6.9 h (FAOS) / 273 h (ICP-MS) Primarily renal excretion (> 50%)
Lobaplatin	Intravenous injection; negligible oral absorption	Low PPB	Not dependent on CYP450 enzyme system	Total Platinum: 6.8 ± 4.3 days Ultrafilterable Platinum: 131 ± 15 min Primarily renal excretion

## Multi-organ toxicity mediated by platinum accumulation

The long-term accumulation of platinum-based agents in normal tissues and organs are core factors for the occurrence of delayed and irreversible chronic toxicity within months to decades after the completion of chemotherapy. Such accumulation-related toxicity can involve multiple systems of the whole body, with clinical manifestations including chronic kidney disease, sensorineural deafness, chronic peripheral neuropathy, refractory anemia, and persistent cancer-related fatigue, which seriously impair the long-term quality of life and even affect the long-term survival outcomes of cancer survivors.

### Nephrotoxicity

The kidney is the primary excretory organ of platinum-based agents, and also the most frequently involved target organ of cisplatin, manifesting as the dual risk of Acute Kidney Injury (AKI) and Chronic Kidney Disease (CKD). 20%–30% of patients receiving high-dose cisplatin will develop AKI, whose core diagnostic features are acute elevation of serum creatinine and rapid decline of Glomerular Filtration Rate (GFR), and up to 40%–100% experience hypomagnesemia (Manohar and Leung 2018); The cumulative incidence of chronic nephrotoxicity reached 37.9% at 3 months after the completion of cisplatin chemotherapy (Kamacı et al. 2022). Long-term follow-up confirms progressive estimated glomerular filtration rate (eGFR) decline and persistent hypomagnesemia as the core manifestations of cisplatin-related long-term nephrotoxicity (Schofield et al. 2024), with risk positively correlated with cumulative dose; high-dose cisplatin increases CKD risk by over threefold in an 18-year cohort (Manohar and Leung 2018).

The cisplatin nephrotoxicity cascade centers on four core links: transporter-mediated specific renal accumulation, mitochondrial damage and oxidative stress, multimodal programmed cell death, and persistent inflammation driving renal interstitial fibrosis.

#### Transporter-mediated renal accumulation

OCT2 and CTR1 are the core transporters mediating the specific uptake and intracellular accumulation of cisplatin in renal proximal tubular epithelial cells. Among them, the transport driving force of OCT2 comes from the transmembrane electrochemical gradient of the substrate. Cisplatin can achieve transmembrane transport through OCT2 via the cell membrane potential difference, which is also the core molecular basis for the specific distribution of cisplatin in tissues and organs such as renal proximal tubules, cochlear hair cells, and dorsal root ganglia, leading to target organ toxicity (Harrach and Ciarimboli 2015). The N-terminal extracellular domain of CTR1 is rich in methionine-enriched motifs. Due to the highly similar coordination chemical structure of divalent hydrated platinum ions (Pt^2^⁺) and copper ions (Cu^2^⁺), Pt^2^⁺ can competitively bind to the methionine binding sites of CTR1 and enter renal tubular epithelial cells through CTR1-mediated endocytosis (Arnesano et al. 2018). Loss-of-function experiments confirmed that CTR1 knockdown can significantly reduce the uptake efficiency of cisplatin by renal tubular epithelial cells, decrease intracellular platinum accumulation, and further inhibit cisplatin-induced renal tubular cell apoptosis (Pabla et al. 2009).

#### Mitochondrial damage and oxidative stress

Active platinum ions entering the cytoplasm of renal tubular epithelial cells can further target and accumulate in mitochondria, which are the core target organelles for intracellular cisplatin accumulation.

Platinum ions enter mitochondria mainly through two pathways: first, the transport and delivery of copper ions (Cu^2^⁺) into mitochondria depend on the copper chaperone protein COX17 (Lutsenko et al. 2025), and platinum ions may enter mitochondria through the COX17-mediated transport pathway via the coordination characteristics similar to Cu^2^⁺ (Zhao et al. 2014); second, hydrated and activated platinum ions are positively charged and can be driven by the − 180 mV transmembrane negative potential of the inner mitochondrial membrane to cross the inner membrane and enter the mitochondrial matrix (Kleih et al. 2019), platinum ions entering mitochondria can bind to mitochondrial DNA (mtDNA) to form stable platinum-mtDNA adducts (Darwish et al. 2017), with the number of adducts being 300–500-fold higher than that of nuclear DNA (Marullo et al. 2013). These stable adducts can continuously disrupt the replication and transcription process of mtDNA and damage the structural integrity of mitochondria, thereby leading to mitochondrial respiratory chain dysfunction: obstruction of the electron transport chain leads to increased electron leakage, and free electrons combine with oxygen molecules to generate superoxide anions (O_2_^⋅^⁻), which further induce the production of a large amount of Reactive Oxygen Species (ROS) and trigger a strong oxidative stress response. Reduced Glutathione (GSH) is the core endogenous antioxidant substance in cells, which can antagonize oxidative damage by scavenging ROS (Su et al. 2023). However, platinum accumulated in cells can covalently bind to the thiol group of GSH, leading to massive depletion of intracellular GSH reserves and collapse of the endogenous antioxidant defense system, which further amplifies the oxidative stress cascade and aggravates mitochondrial damage (Wang et al. 2023), ultimately triggering multiple forms of regulated cell death, including apoptosis, necroptosis, and pyroptosis, in renal tubular epithelial cells (Tang et al. 2023).

#### Persistent inflammatory immune disorder and renal interstitial fibrosis

**Fig. 2:**
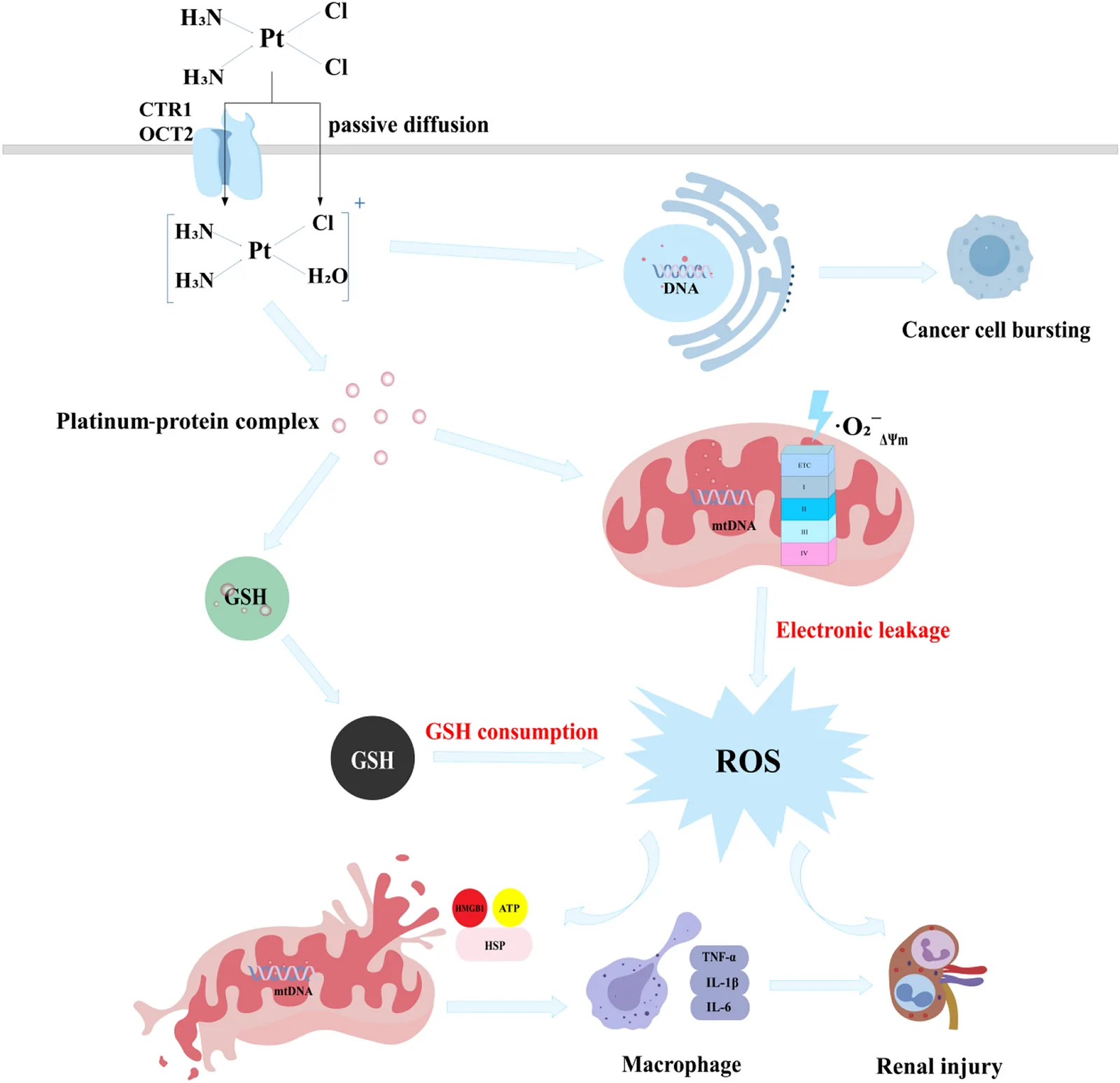
The mechanism of toxicity caused by the accumulation of platinum complex

After programmed cell death of renal tubular epithelial cells, the integrity of the cell membrane is destroyed, and a large amount of Damage-Associated Molecular Patterns (DAMPs) are released. DAMPs can bind to and activate pattern recognition receptors on the surface of renal innate immune cells (such as macrophages and dendritic cells) and renal tubular epithelial cells, thereby activating downstream core pro-inflammatory signaling pathways such as NF-κB, and inducing the massive secretion of pro-inflammatory factors including Tumor Necrosis Factor-α (TNF-α), Interleukin-1β (IL-1β), and Interleukin-6 (IL-6) (Fu et al. 2023), forming a positive feedback persistent inflammatory cascade. Meanwhile, long-term intracellular accumulation of platinum ions and persistent oxidative stress can activate the senescence program of renal tubular epithelial cells, induce cell cycle arrest and Senescence-Associated Secretory Phenotype (SASP), release a large number of pro-inflammatory and pro-fibrotic factors through paracrine effects, further amplify the local inflammatory response, and activate the proliferation and activation of renal interstitial fibroblasts (Nakao et al. 2024) . The continuous progression of the above pathological processes ultimately leads to renal tubular atrophy, renal interstitial fibrosis, and progressive irreversible decline of renal function, which is the core pathological mechanism of cisplatin-induced AKI to CKD progression (Fig.2) (Li et al. 2025; Manohar and Leung 2018).

### Neurotoxicity and ototoxicity

The nervous system is a frequently involved target organ for the cumulative toxicity of platinum-based agents. There are significant differences in the neurotoxic target sites, clinical phenotypes, and accumulation potential of different platinum-based agents, among which oxaliplatin-mediated peripheral sensory neuropathy and cisplatin-mediated irreversible ototoxicity are the most typical.

#### Peripheral sensory neuropathy

Oxaliplatin has the highest incidence of neurotoxicity among clinical platinum-based agents, and its mediated neurotoxicity has a characteristic biphasic phenotype, which is divided into acute cold-triggered neural hypersensitivity and chronic cumulative sensory neuropathy.

Acute neurotoxicity occurs within hours of administration, triggered by cold exposure, manifesting as transient paresthesia/numbness in the extremities, perioral region and throat, often with muscle or laryngospasm. Approximately 89% of patients develop symptoms in the first chemotherapy cycle, which peak within 3 days and mostly resolve spontaneously (Pachman et al. 2015).

Long-term specific platinum accumulation in nerve tissues is the core driver of oxaliplatin-induced chronic neuropathy. In colorectal cancer patients treated with the FOLFOX regimen (fluorouracil, calcium folinate combined with oxaliplatin), platinum residues remain detectable in dermal collagen fibers up to 60 months post-withdrawal, mediating persistent, distally distributed neuropathy via damaging cutaneous sensory nerve fibers (Cao et al. 2019). The Dorsal root ganglion (DRG) is the core target tissue of oxaliplatin neurotoxicity. Its structural feature of lacking the blood-nerve barrier leads to the circulating platinum-diaminocyclohexane (DACH) complex being mediated by OCT2 (Huang et al. 2020) to accumulate in large quantities specifically in the DRG (Wei et al. 2021). Accumulated platinum ions can induce oxidative stress damage and activate the neuroinflammatory cascade, leading to DRG neuron damage, axonal degeneration, and nerve conduction disorders, and ultimately trigger chronic sensory neuropathy (Lin et al. 2025; Zhang et al. 2024; Calls et al. 2022). Animal studies confirm oxaliplatin can accumulate long-term in the DRG, spinal cord and even the central nervous system, mediating chronic pain and anxiety-like behaviors (Reis et al. 2024). The differences in peripheral neurotoxicity characteristics, accumulation potential, and incidence risk of different platinum-based agents are summarized in Table 2 (McWhinney et al. 2009; Lazić et al. 2020; Avan et al. 2015; Pignata et al. 2006; Zhang et al. 2017; Wang et al. 2018; Yang et al. 2021).

**Table 2:** Differences in neurotoxicity caused by platinum-based drugs

Drug	Neurotoxicity characteristics	Incidence & severity	Typical symptoms	Mechanisms & risk factors	Prevention & management strategies
Cisplatin	Chronic, cumulative; predominantly sensory; slow recovery over months to years, potentially irreversible	High (Ototoxicity: 75–100%)	Persistent limb numbness, reduced vibration/temperature sensation, occasional hearing loss	Selective accumulation in DRG, leading to large-diameter sensory neuron degeneration	Monitor cognition & peripheral neuropathy; avoid high-dose prolonged use/neurotoxic drug co-administration. Amifostine, vitamin E, glutathione have potential unproven neuroprotection
Carboplatin	Primarily peripheral sensory neuropathy	Low (4–6%)	Similar to cisplatin but milder and more slowly progressive; stronger correlation with cumulative dose	Higher risk in ≥ 65 years / prior cisplatin use; third-generation agents (paclitaxel/gemcitabine) nearly double neurotoxicity risk	Dose adjustment or treatment interruption; strict assessment of residual neuropathy before retreatment. Limited protective effect of amifostine and glutathione
Nedaplatin	Mainly peripheral neuropathy	Significantly lower than cisplatin	Cramps, headache, cold extremities; mostly mild to moderate (Grade 1–2), no significant severe neurotoxicity	NA	NA
Oxaliplatin	Acute and chronic forms coexist	High (70% sensory neuropathy, 50% chronic type)	Acute: Cold sensitivity, laryngospasm, transient numbness Chronic: Distal acral sensory abnormalities, intact motor conduction	Chronic: Cisplatin-like; motor axons insensitive; RBC membrane protein binding reduces free platinum & neuronal damage	Advise patients to avoid cold stimuli during treatment; closely monitor progression of sensory abnormalities. Duloxetine significantly reduces pain and paresthesia
Lobaplatin	Peripheral neurotoxicity; generally reversible	Few cases of mild sensory disturbances; only mild toxicity, no moderate or severe reactions reported	Acral numbness, tingling	NA	NA

#### Ototoxicity and irreversible hearing impairment

In addition to peripheral neuropathy, long-term platinum accumulation in cochlear tissues is the core driver of delayed, irreversible post-chemotherapy ototoxicity. Residual serum platinum levels are significantly positively correlated with ototoxicity severity 5–20 years after cisplatin treatment (Sprauten et al. 2012), with cisplatin-treated patients having a markedly higher high-frequency hearing threshold than chemotherapy-naive controls (Skalleberg et al. 2017). Cisplatin-mediated ototoxicity is dose-dependent, bilaterally symmetric, progressive and irreversible, starting with high-frequency hearing loss and extending to mid/low frequencies with increasing accumulation; over 50% of patients have persistent tinnitus, with a far higher incidence than carboplatin and oxaliplatin. Each 100 mg/m^2^ increase in cumulative dose (mg/m^2^ is the standard clinical dosing unit of platinum-based agents based on the patient's Body Surface Area, BSA) raises hearing impairment risk by 5%–7%, and prior noise exposure triples ototoxicity risk (Waissbluth et al. 2017; Wei et al. 2021). Carboplatin has minimal ototoxicity at conventional doses (risk elevated only at ≥ 2000 mg/m^2^ or intrathecal administration) (McKeage 1995), while oxaliplatin, nedaplatin and lobaplatin have weak cochlear affinity and rare ototoxicity (Hellberg et al. 2009). Pediatric patients < 5 years old are the highest-risk group for permanent hearing loss due to developing cochlear structures, requiring mandatory hearing monitoring during chemotherapy (Langer et al. 2013).

The Stria Vascularis (SV) is the core accumulation site of platinum in cochlear tissues, where platinum can be retained for months to years. In human cochlear tissues, cisplatin can be retained for at least 18 months, and the concentration is the highest in the stria vascularis region, which is 2–3 times that of the organ of Corti and spiral ganglion. This is also the core mechanism of cisplatin-mediated delayed hearing loss (Breglio et al. 2017). Long-term accumulation of cisplatin in the stria vascularis can directly damage the structure of the stria vascularis, leading to increased permeability of the Blood-Labyrinth Barrier (BLB), decreased endolymphatic potassium ion concentration, and decreased endocochlear potential, destroying the endolymphatic ion homeostasis. At the same time, it activates the inflammatory response of outer hair cells, further promoting the entry of cisplatin into the endolymph, forming a vicious cycle of accumulation and damage (Wang et al. 2023). Cisplatin entering the endolymph can enter cochlear hair cells through transporters such as CTR1 and OCT2, and accumulate in large quantities in the mitochondria of hair cells mediated by the copper chaperone protein COX17 (Peng et al. 2025), destroying the structure and function of mitochondria, causing energy metabolism disorders, and then inducing the generation of a large amount of ROS and persistent neuroinflammatory response (Tan and Vlajkovic 2023), ultimately leading to multimodal programmed cell death of cochlear hair cells and spiral ganglion neurons (Wang et al. 2023). OCT2 inhibitors (e.g., lansoprazole) significantly reduce cochlear platinum accumulation and protective against cisplatin ototoxicity in animal models (Wang et al. 2023). The second-generation nedaplatin and third-generation lobaplatin have weak cochlear accumulation capacity, and the risk of ototoxicity is significantly lower than that of cisplatin.

### Immunotoxicity and immune microenvironment disorder

Platinum-based agents enhance anti-tumor immune response by inducing Immunogenic Cell Death (ICD), enhancing the killing sensitivity of tumor cells to cytotoxic T cells, and improving the immunosuppressive microenvironment. Based on these immune-modulatory mechanisms, the combination therapy with immune checkpoint inhibitors in clinical practice has shown synergistic efficacy in solid tumors such as non-small cell lung cancer and colorectal cancer, providing a new strategy for tumor treatment (Lesterhuis et al. 2011; Hato et al. 2014; de Biasi et al. 2014; Rébé et al. 2019).

However, platinum-based treatment is also accompanied by immune-related toxicities. In addition to acute myelosuppression-induced hematopoietic and innate immunity impairment, the main upstream mechanism of immune toxicity caused by platinum element accumulation may be the continuous release of pro-inflammatory factors due to oxidative stress and the occurrence of systemic chronic low-level inflammation (Domingo et al. 2022).

#### Regulation of systemic immune cell subsets function

CD28 is an essential “second signal” for T cell activation, and its loss of expression can directly lead to T cell entering an immune anergy state (Alegre et al. 2001). CD4⁺CD28⁻ T cells (linked to immune senescence, pro-inflammatory and cytotoxicity) (Maly and Schirmer 2015) and CD8⁺CD28⁻ T cells (elevated in chronic inflammation/senescence, immunosuppressive and cytotoxicity) (Arosa 2002) are significantly increased in the peripheral blood of platinum-treated lung cancer patients 30 days post-chemotherapy, which may be associated with impaired T cell function and tumor immune surveillance (Saavedra et al. 2016). In addition, by comparing the influenza vaccine immune response of patients treated with platinum-based dual-drug combination chemotherapy and single-drug chemotherapy, it was confirmed that platinum-based chemotherapy can directly inhibit the antibody secretion function of B cells and significantly weaken the immunogenicity and protective efficacy of the vaccine (Nakashima et al. 2017). Persistent impairment of immune function can further lead to the decline of the body's immune defense capacity. Clinical data show that when using docetaxel combined with carboplatin regimen in the treatment of non-small cell lung cancer, the incidence of febrile neutropenia in elderly patients ≥ 65 years old (7.0%) is significantly higher than that in patients < 65 years old (2.4%), and the risk of grade 3–4 infection is higher (Schuette et al. 2011).

#### Tumor microenvironment remodeling for tumor progression and chemoresistance

Long-term platinum accumulation in the tumor microenvironment drives the formation of a local immunosuppressive microenvironment, tumor progression, and chemoresistance.

Accumulated platinum activates the p38 mitogen-activated protein kinase (p38 MAPK)—a key stress and inflammatory signaling kinase, and NF-κB signaling pathways in dendritic cells (DCs), promoting secretion of immunosuppressive IL-10 and inducing tumor immune escape (Kim et al. 2016).

Notably, platinum can accumulate massively and persistently in cancer-associated fibroblasts (CAFs) (Chelushkin et al. 2024), activating the TGF-β pathway to induce secretion of pro-tumor factors (IL-11, periostin) (Linares et al. 2023), which drives tumor cell epithelial-mesenchymal transition, immune escape, invasion/metastasis, and paracrine-mediated chemoresistance (Chen and Chang 2019). Platinum accumulation also induces tumor cell senescence and a TGF-β-centered senescence-associated secretory phenotype (SASP), which promotes tumor progression and chemoresistance (González-Gualda et al. 2026).

#### Hypersensitivity reactions

Another important adverse effect of platinum-based agents on the immune system is IgE-mediated type I hypersensitivity. Platinum ions act as haptens, binding to serum proteins to form complete antigens and induce specific IgE production; re-exposure triggers mast cell/basophil degranulation, releasing inflammatory mediators and causing urticaria, bronchospasm, hypotension, or even anaphylactic shock (Sliesoraitis and Chikhale 2005). Hypersensitivity risk is closely linked to drug type, exposure duration, and cumulative dose: carboplatin hypersensitivity is common in patients with cumulative dose > 650 mg; oxaliplatin hypersensitivity mostly occurs after the 6th infusion (overall incidence ~ 18.9%, rare severe reactions); cisplatin has a low overall incidence, but risk rises with concurrent radiotherapy (Makrilia et al. 2010). Notably, serum platinum concentration is positively correlated with hypersensitivity risk, with each 1 mg/L increase linked to a 0.007-fold higher allergy risk, indicating that long-term platinum accumulation aggravates systemic inflammation and hypersensitivity via persistent immune cell interference (Zhang et al. 2020).

To date, studies on the immunotoxicity mechanisms of platinum accumulation remain scarce. Although Pb, Cd and Pt differ in their toxicokinetic profiles, they are all divalent heavy metals and may share common mechanisms. It has been well documented that Pb accumulation triggers oxidative stress via excessive ROS production and GSH depletion, which impairs immune cell function and drives a Th2-biased immune response (Fenga et al. 2017). For Cd, it disrupts the mitochondrial respiratory chain to induce excessive ROS generation, coupled with GSH depletion, CD4⁺ T cell apoptosis and a reduced CD4⁺/CD8⁺ ratio, disrupt the adaptive immune homeostasis of the body (Ebrahimi et al. 2020; Lee et al. 2019; Rowley and Monestier 2005). It provides reasonable support for the core hypothesis we proposed, which is that the accumulation of platinum leads to immune system disorders in the body through the chronic inflammatory microenvironment mediated by oxidative stress.

### Cancer-related fatigue mediated by platinum accumulation

Cancer-Related Fatigue (CRF) is one of the most common long-term adverse reactions in tumor patients after chemotherapy, characterized by rest-unrelieved persistent fatigue and reduced activity tolerance that severely impairs cancer survivors’ quality of life (Modak et al. 2017). Long-term accumulation of platinum in the body is an important risk factor for the occurrence of CRF. Clinical data show 57% of cisplatin-treated ovarian cancer patients have moderate-to-severe fatigue, with 27% still experiencing unrelieved symptoms 18 months post-chemotherapy (Beesley et al. 2020). Platinum accumulation mediates the occurrence of CRF mainly through three core pathways: chronic inflammatory response, anemia, and mitochondrial dysfunction.

#### Chronic inflammatory response

**Fig. 3:**
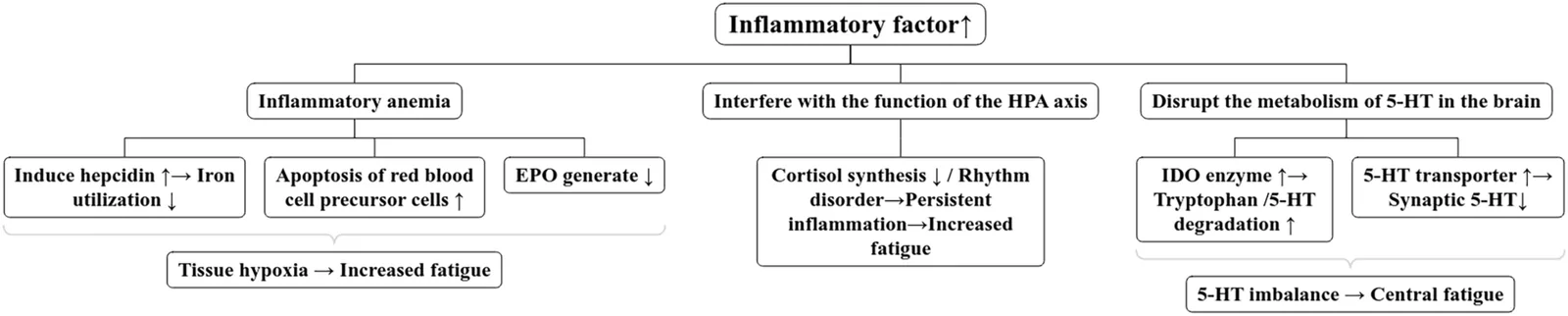
The mechanism of fatigue caused by pro-inflammatory factors

Long-term accumulation of platinum in multiple tissues can induce the generation of a large amount of ROS, promote the sustained release of pro-inflammatory cytokines such as TNF-α, IL-6, and IL-1β, and form a systemic chronic low-grade inflammatory microenvironment, which is the core driving mechanism of CRF (Bower and Lamkin 2013). Elevated pro-inflammatory cytokines may directly trigger fatigue via inflammatory anemia (Weiss et al. 2019; Fraenkel 2016; Zhang et al. 2021), hypothalamic–pituitary–adrenal (HPA) axis and the 5-hydroxytryptamine (5-HT) metabolic pathway (Fig. 3) (Bower 2014; Turnbull and Rivier 1995; Bower and Lamkin 2013). Clinical evidence confirms anti-IL-6 targeted therapy rapidly alleviates CRF, while anemia correction with erythropoietin (EPO) alone has limited efficacy, further validating the central role of chronic inflammation (Modak et al. 2017; Jager et al. 2008).

#### Platinum-related anemia

Anemia is an important synergistic risk factor for CRF, and platinum can mediates anemia through two major pathways: first, cisplatin and carboplatin have strong myelotoxicity, which can directly damage bone marrow erythroid progenitor cells, inhibit their proliferation and differentiation, leading to decreased erythropoiesis (Bryer and Henry 2018); second, cisplatin can directly inhibit the physiological function of human iron regulatory protein 2 by covalently binding to its cysteine residue sites (Cys512 and Cys516), disrupting the homeostasis of systemic iron metabolism, causing persistent intracellular iron deficiency, and further aggravating refractory anemia (Miyazawa et al. 2019). Anemia-induced tissue hypoxia worsens skeletal muscle energy metabolism disorders, synergistically amplifying CRF with chronic inflammation.

#### Mitochondrial dysfunction

Mitochondria are the core organelles of cellular energy metabolism, and mitochondrial energy metabolism defects have been confirmed to be an important potential inducer of CRF (Zhang and Perry 2024). Cisplatin accumulates persistently in the mitochondria of energy metabolism-related organs (skeletal muscle, liver), forms platinum-mtDNA adducts to disrupt mitochondrial structure and function, and causes oxidative phosphorylation impairment and insufficient ATP production, ultimately driving persistent fatigue (Huot et al. 2023). Recent studies further identify the GDF15-GFRAL axis as an additional pathway linking mitochondrial dysfunction to cisplatin-related CRF (Chelette et al. 2023).

## Other long-term toxicities

Beyond the aforementioned target organ toxicities, long-term cohort studies have confirmed that platinum-based chemotherapy induces multiple delayed long-term adverse outcomes (manifesting years to decades post-chemotherapy, severely impairing cancer survivors’ long-term survival), including reproductive toxicity, cardiovascular toxicity, and elevated second primary neoplasm (SPN) risk. Cisplatin preferentially accumulates in gonadal tissues, causing irreversible reproductive damage: cisplatin cycle number is negatively correlated with long-term fertility in male testicular cancer survivors (Solheim et al. 2015), and platinum-based chemotherapy drives sustained depletion of immature testicular germ cells, potentially leading to permanent infertility (Tharmalingam et al. 2020). Platinum retained in vascular tissue for up to 30 years causes persistent endothelial damage, elevating long-term vascular disease risk (Herrmann 2020); 20-year follow-up revealed accelerated vascular aging in cisplatin-treated survivors (Stelwagen et al. 2020), with a cumulative dose ≥ 200 mg/m^2^ is linked to persistent biventricular systolic dysfunction and myocardial tissue alterations (Beitzen-Heineke et al. 2024). Long-term platinum retention also drives sustained SPN risk, a large-scale cohort of 5625 testicular cancer survivors showed a sustained, significant increase in SPN risk among cisplatin-treated patients, with a 1.6–2.1-fold higher risk in those receiving ≥ 2 cycles of cisplatin versus chemotherapy-naive patients, particularly for small intestinal and bladder cancers. This is potentially driven by decades-long plasma platinum retention and months-long tissue platinum sequestration, which sustain platinum–DNA adduct formation, genomic instability and oncogenic mutations (Hellesnes et al. 2020). In pediatric cancer survivors, platinum-based chemotherapy induces persistent, characteristic somatic mutation accumulation in peripheral blood cells, which may underlie the long-term risk of secondary leukemia (Ueda et al. 2022).

However, the direct causal relationship and molecular mechanism between platinum accumulation and the above long-term toxicities have not been fully elucidated, which still needs to be further verified by large-sample cohort studies and basic mechanism studies in the future.

## Clinical challenges and mitigation strategies

### Core clinical and research challenges

With the widespread clinical use of platinum-based chemotherapeutics, long-term in vivo platinum accumulation has emerged as a critical yet underrecognized clinical challenge, serving as the core toxicological driver of impaired long-term quality of life and adverse health outcomes in cancer survivors. However, research in this field currently faces multiple obstacles.

Most clinical assays only measure total platinum levels, while the toxicological activity varies drastically among platinum species with distinct chemical forms (e.g., inert complexes bound to GSH, MT, and methionine), leading to inaccurate assessment of accumulation-related toxicity risk.

Marked interindividual heterogeneity exists in platinum accumulation and toxicity severity among patients with similar cumulative doses, which is mainly mediated by genetic polymorphisms (e.g., GSTP1), epigenetic modifications, and baseline health status. This poses a major barrier to establishing an individualized dose-accumulation-toxicity correlation model.

There is a lack of reliable, sensitive biomarkers that are directly linked to platinum accumulation for early prediction of delayed long-term toxicities (e.g., renal fibrosis, chronic peripheral neuropathy).

Clinically validated individualized platinum elimination regimens are unavailable, and targeted chelation therapy for accumulated platinum remains in the exploratory stage, with unconfirmed efficacy and safety.

### Targeted mitigation strategies

#### Therapeutic monitoring and individualized dosing optimization

Continuous therapeutic drug monitoring (TDM) throughout chemotherapy cycles is recommended, combined with early renal toxicity biomarkers (β2-microglobulin, kidney injury molecule-1 [KIM-1], trefoil factor 3 [TFF3]) (George et al. 2017) to enable early warning of accumulation-related toxicity. Genotyping of key genes regulating platinum metabolism and accumulation (e.g., GSTP1) and DNA damage repair (e.g., ERCC1, BRCA1/2) should be performed to screen patients at high risk of excessive accumulation and severe toxicity, as well as those most likely to benefit from platinum-based regimens. Pharmacokinetic/pharmacodynamic models should be applied to dynamically adjust platinum dosing, especially in elderly or renally impaired patients, to minimize excessive platinum accumulation while preserving anti-tumor efficacy.

#### Nutritional and lifestyle adjuvant interventions

Increased intake of antioxidant-rich foods (vitamin C/E, β-carotene, selenium) and dietary fiber is recommended to alleviate platinum accumulation-induced oxidative damage; selenium, as a coenzyme of GSH peroxidase, can also promote platinum metabolism and reduce its in vivo retention. Zinc and magnesium supplementation can correct electrolyte disorders caused by platinum-based agents. High-salt, high-fat, and spicy irritant foods should be restricted. For oxaliplatin-treated patients, strict cold protection is mandatory to avoid cold-induced exacerbation of neurotoxicity. Moderate exercise, stress management, and adequate sleep are encouraged to improve overall treatment tolerance.

#### Analytical technology development and targeted chelation therapy

Further development of in situ analytical techniques is required to characterize the chemical speciation and subcellular distribution of accumulated platinum, which is the basis for accurate toxicity assessment and targeted intervention. Given the conserved chelating properties of transition metal complexes, existing clinical metal chelators can be repurposed and optimized for targeted platinum chelation, with priority given to strategies that reduce platinum accumulation in normal tissues while preserving intratumoral platinum concentrations. Future studies can develop targeted nanocarriers loaded with platinum-specific chelators or adsorbents to achieve localized detoxification and minimize systemic side effects; platinum-binding proteins (e.g., albumin) can also be leveraged to design targeted delivery systems, which can simultaneously serve as biomarkers for platinum accumulation monitoring (Tao et al. 2021).

#### Translational research of accumulation-related toxicity biomarkers

Clinical translation of candidate biomarkers directly linked to platinum accumulation should be prioritized for early prediction of long-term toxicities, including mitochondrial function markers (TFAM, TIMM23), cellular senescence marker p16 (Li et al. 2023), oxidative stress marker 8-isoprostane, and ototoxicity-related miRNAs (miR-214 and miR-182) (Quintanilha et al. 2019).

## Limitations of this review

This review systematically summarizes the pharmacokinetic basis, multi-organ toxicity mechanisms, chemoresistance link, clinical challenges, and intervention strategies of in vivo platinum accumulation from platinum-based chemotherapeutics.

However, there are still some limitations: the causal chain between platinum accumulation and delayed target organ toxicity is not fully delineated, with insufficient in-depth analysis of organ-specific toxic mechanisms; the heterogeneous toxic effects of platinum species with different binding forms and subcellular distributions are not systematically elaborated, and the comparative analysis of accumulation kinetics, target organ tropism, and toxicity risk of clinical platinum agents is incomplete; some conclusions on platinum accumulation and long-term complications are only based on observational cohort data, with unadjusted confounding factors, leaving the causal evidence chain to be further improved.

These limitations reflect both the shortcomings of this review constrained by existing evidence, and the urgent core scientific questions and unmet clinical needs in the toxicology and clinical application of platinum-based agents. Future interdisciplinary efforts, including in-depth mechanistic studies, large multicenter long-term follow-up trials, and translational research on precise intervention targets, are needed to address these gaps.

## Data Availability

No datasets were generated or analysed during the current study.

## References

[CR1] Abd Elnabi MK, Elkaliny NE, Elyazied MM, Azab SH, Elkhalifa SA, Elmasry S, Mouhamed MS, Shalamesh EM, Alhorieny NA, Abd Elaty AE (2023) Toxicity of heavy metals and recent advances in their removal: a review. Toxics 11:580. 10.3390/toxics11070580 37505546 10.3390/toxics11070580 PMC10384455

[CR2] Alassadi S, Pisani MJ, Wheate NJ (2022) A chemical perspective on the clinical use of platinum-based anticancer drugs. Dalton Trans 51:10835–10846. 10.1039/D2DT01875F 35781551 10.1039/d2dt01875f

[CR3] Alegre M-L, Frauwirth KA, Thompson CB (2001) T-cell regulation by CD28 and CTLA-4. Nat Rev Immunol 1:220–228. 10.1038/35105024 11905831 10.1038/35105024

[CR4] Arnesano F, Nardella MI, Natile G (2018) Platinum drugs, copper transporters and copper chelators. Coord Chem Rev 374:254–260. 10.1016/j.ccr.2018.07.003

[CR5] Arosa FA (2002) CD8+ CD28–T cells: certainties and uncertainties of a prevalent human T-cell subset. Immunol Cell Biol 80:1–13. 10.1046/j.1440-1711.2002.01057.x 11869357 10.1046/j.1440-1711.2002.01057.x

[CR6] Avan A, Postma TJ, Ceresa C, Avan A, Cavaletti G, Giovannetti E, Peters GJ (2015) Platinum-induced neurotoxicity and preventive strategies: past, present, and future. Oncologist 20:411–432. 10.1634/theoncologist.2014-0044 25765877 10.1634/theoncologist.2014-0044 PMC4391771

[CR7] Beesley VL, Webber K, Nagle CM, DeFazio A, Obermair A, Williams M, Friedlander M, Webb PM, OPAL Study Group (2020) When will I feel normal again? Trajectories and predictors of persistent symptoms and poor wellbeing after primary chemotherapy for ovarian cancer. Gynecol Oncol 159:179–186. 10.1016/j.ygyno.2020.07.029 32773150 10.1016/j.ygyno.2020.07.029

[CR8] Begerow J (1999) Long term urinary platinum, palladium, and gold excretion of patients after insertion of noble metal dental alloys. Biomarkers 4:27–36. 10.1080/135475099230976 23898792 10.1080/135475099230976

[CR9] Beitzen-Heineke A, Rolling CC, Seidel C, Erley J, Molwitz I, Muellerleile K, Saering D, Senftinger J, Börschel N, Engel NW (2024) Long-term cardiotoxicity in germ cell cancer survivors after platinum-based chemotherapy: cardiac MR shows impaired systolic function and tissue alterations. Eur Radiol 34:4102–4112. 10.1007/s00330-023-10420-w 37982836 10.1007/s00330-023-10420-w PMC11166766

[CR10] Benedetti BT, Peterson EJ, Kabolizadeh P, Martínez A, Kipping R, Farrell NP (2011) Effects of noncovalent platinum drug–protein interactions on drug efficacy: use of fluorescent conjugates as probes for drug metabolism. Mol Pharm 8:940–948. 10.1021/mp2000583 21548575 10.1021/mp2000583 PMC3341405

[CR11] Bharti R, Sharma R (2022) Effect of heavy metals: an overview. Mater Today Proc 51:880–885. 10.1016/j.matpr.2021.06.278

[CR12] Bower JE (2014) Cancer-related fatigue—mechanisms, risk factors, and treatments. Nat Rev Clin Oncol 11:597–609. 10.1038/nrclinonc.2014.127 25113839 10.1038/nrclinonc.2014.127 PMC4664449

[CR13] Bower JE, Lamkin DM (2013) Inflammation and cancer-related fatigue: mechanisms, contributing factors, and treatment implications. Brain Behav Immun 30:S48–S57. 10.1016/j.bbi.2012.06.011 22776268 10.1016/j.bbi.2012.06.011 PMC3978020

[CR14] Breglio AM, Rusheen AE, Shide ED, Fernandez KA, Spielbauer KK, McLachlin KM, Hall MD, Amable L, Cunningham LL (2017) Cisplatin is retained in the cochlea indefinitely following chemotherapy. Nat Commun 8:1654. 10.1038/s41467-017-01837-1 29162831 10.1038/s41467-017-01837-1 PMC5698400

[CR15] Brouwers EEM, Huitema ADR, Beijnen JH, Schellens JHM (2008) Long-term platinum retention after treatment with cisplatin and oxaliplatin. BMC Clin Pharmacol 8:1–10. 10.1186/1472-6904-8-7 18796166 10.1186/1472-6904-8-7 PMC2559818

[CR16] Bryer E, Henry D (2018) Chemotherapy-induced anemia: etiology, pathophysiology, and implications for contemporary practice. Int J Clin Transfus Med 21–31. 10.2147/ijctm.s187569

[CR17] Budi HS, Opulencia MJC, Afra A, Abdelbasset WK, Abdullaev D, Majdi A, Taherian M, Ekrami HA, Mohammadi MJ (2024) Source, toxicity and carcinogenic health risk assessment of heavy metals. Rev Environ Health 39:77–90. 10.1515/reveh-2022-0096 36181731 10.1515/reveh-2022-0096

[CR18] Calls A, Torres‐Espin A, Tormo M, Martínez‐Escardó L, Bonet N, Casals F, Navarro X, Yuste VJ, Udina E, Bruna J (2022) A transient inflammatory response contributes to oxaliplatin neurotoxicity in mice. Ann Clin Transl Neurol 9:1985–1998. 10.1002/acn3.51691 36369764 10.1002/acn3.51691 PMC9735376

[CR19] Cao Y, Chang Q, Zhang W, Ornatsky O, Hedley D, Chen EX (2019) Skin platinum deposition in colorectal cancer patients following oxaliplatin-based therapy. Cancer Chemother Pharmacol 84:1195–1200. 10.1007/s00280-019-03956-6 31520102 10.1007/s00280-019-03956-6

[CR20] Chelette B, Chidomere CL, Dantzer R (2023) The GDF15-GFRAL axis mediates chemotherapy-induced fatigue in mice. Brain Behav Immun 108:45–54. 10.1016/j.bbi.2022.11.008 36427806 10.1016/j.bbi.2022.11.008 PMC9868083

[CR21] Chelushkin MA, van Dorp J, van Wilpe S, Seignette IM, Mellema J-J, Alkemade M, Gil-Jimenez A, Peters D, Brugman W, Stockem CF (2024) Platinum-based chemotherapy induces opposing effects on immunotherapy response-related spatial and stromal biomarkers in the bladder cancer microenvironment. Clin Cancer Res 30:4227–4239. 10.1158/1078-0432.CCR-24-0724 39047168 10.1158/1078-0432.CCR-24-0724

[CR22] Chen S-H, Chang J-Y (2019) New insights into mechanisms of cisplatin resistance: from tumor cell to microenvironment. Int J Mol Sci 20:4136. 10.3390/ijms20174136 31450627 10.3390/ijms20174136 PMC6747329

[CR23] Colombo C, Oates CJ, Monhemius AJ, Plant JA (2008) Complexation of platinum, palladium and rhodium with inorganic ligands in the environment. Geochem Explor Environ Anal 8:91–101. 10.1144/1467-7873/07-151

[CR24] Corinti D, Crestoni ME, Chiavarino B, Fornarini S, Scuderi D, Salpin J-Y (2020) Insights into cisplatin binding to uracil and thiouracils from IRMPD spectroscopy and tandem mass spectrometry. J Am Soc Mass Spectrom 31:946–960. 10.1021/jasms.0c00006 32233383 10.1021/jasms.0c00006 PMC7997577

[CR25] Darwish MA, Abo‐Youssef AM, Khalaf MM, Abo‐Saif AA, Saleh IG, Abdelghany TM (2017) Vitamin E mitigates cisplatin‐induced nephrotoxicity due to reversal of oxidative/nitrosative stress, suppression of inflammation and reduction of total renal platinum accumulation. J Biochem Mol Toxicol 31:1–9. 10.1002/jbt.21833 27550472 10.1002/jbt.21833

[CR26] de Biasi AR, Villena-Vargas J, Adusumilli PS (2014) Cisplatin-induced antitumor immunomodulation: a review of preclinical and clinical evidence. Clin Cancer Res 20:5384–5391. 10.1158/1078-0432.CCR-14-1298 25204552 10.1158/1078-0432.CCR-14-1298 PMC4216745

[CR27] Domingo IK, Latif A, Bhavsar AP (2022) Pro-inflammatory signalling PRRopels cisplatin-induced toxicity. Int J Mol Sci 23:7227. 10.3390/ijms23137227 35806229 10.3390/ijms23137227 PMC9266867

[CR28] Ebrahimi M, Khalili N, Razi S, Keshavarz-Fathi M, Khalili N, Rezaei N (2020) Effects of lead and cadmium on the immune system and cancer progression. J Environ Health Sci Eng 18:335–343. 10.1007/s40201-020-00455-2 32399244 10.1007/s40201-020-00455-2 PMC7203386

[CR29] Ek KH, Rauch S, Morrison GM, Lindberg P (2004) Platinum group elements in raptor eggs, faeces, blood, liver and kidney. Sci Total Environ 334:149–159. 10.1016/j.scitotenv.2004.04.067 15504501 10.1016/j.scitotenv.2004.04.067

[CR30] Esteban-Fernández D, Verdaguer JM, Ramirez-Camacho R, Palacios MA, Gómez-Gómez MM (2008) Accumulation, fractionation, and analysis of platinum in toxicologically affected tissues after cisplatin, oxaliplatin, and carboplatin administration. J Anal Toxicol 32:140–146. 10.1093/jat/32.2.140 18334097 10.1093/jat/32.2.140

[CR31] Fenga C, Gangemi S, Di Salvatore V, Falzone L, Libra M (2017) Immunological effects of occupational exposure to lead. Mol Med Rep 15:3355–3360. 10.3892/mmr.2017.6381 28339013 10.3892/mmr.2017.6381

[CR32] Fraenkel PG (2016) Anemia of inflammation: a review. Med Clin North Am 101:285. 10.1016/j.mcna.2016.09.005 28189171 10.1016/j.mcna.2016.09.005 PMC5308549

[CR33] Fu Y, Xiang Y, Wang Y, Liu Z, Yang D, Zha J, Tang C, Cai J, Chen G, Dong Z (2023) The STAT1/HMGB1/NF-κB pathway in chronic inflammation and kidney injury after cisplatin exposure. Theranostics 13:2757. https://www.thno.org/v13p2757.htm 37284446 10.7150/thno.81406 PMC10240827

[CR34] Gagnon ZE, Newkirk C, Hicks S (2006) Impact of platinum group metals on the environment: a toxicological, genotoxic and analytical chemistry study. J Environ Sci Health A Tox Hazard Subst Environ Eng 41:397–414. 10.1080/10934520500423592 16484072 10.1080/10934520500423592

[CR35] George B, Wen X, Mercke N, Gomez M, O’Bryant C, Bowles DW, Hu Y, Hogan SL, Joy MS, Aleksunes LM (2017) Profiling of kidney injury biomarkers in patients receiving cisplatin: time‐dependent changes in the absence of clinical nephrotoxicity. Clin Pharmacol Ther 101:510–518. 10.1002/cpt.606 28002630 10.1002/cpt.606 PMC5359028

[CR36] González-Gualda E, Reinius MA, Macias D, Morsli S, Ge J, Olan I, Martín JE, Ou HL, Hartono M, Puerto-Camacho MP, Denholm M (2026) Treatment resistance to platinum-based chemotherapy in lung and ovarian cancer is driven by a targetable TGFβ senescent secretome. Nat Aging 1–25. 10.1038/s43587-025-01054-2 10.1038/s43587-025-01054-2 PMC12920146 41634464

[CR37] Graham MA, Lockwood GF, Greenslade D, Brienza S, Bayssas M, Gamelin E (2000) Clinical pharmacokinetics of oxaliplatin: a critical review. Clin Cancer Res 6:1205–1218 10778943

[CR38] Hall MD, Okabe M, Shen D-W, Liang X-J, Gottesman MM (2008) The role of cellular accumulation in determining sensitivity to platinum-based chemotherapy. Annu Rev Pharmacol Toxicol 48:495–535. 10.1146/annurev.pharmtox.48.080907.180426 17937596 10.1146/annurev.pharmtox.48.080907.180426

[CR39] Harrach S, Ciarimboli G (2015) Role of transporters in the distribution of platinum-based drugs. Front Pharmacol 6:85. 10.3389/fphar.2015.00085 25964760 10.3389/fphar.2015.00085 PMC4408848

[CR40] Hato SV, Khong A, de Vries IJM, Lesterhuis WJ (2014) Molecular pathways: the immunogenic effects of platinum-based chemotherapeutics. Clin Cancer Res 20:2831–2837. 10.1158/1078-0432.CCR-13-3141 24879823 10.1158/1078-0432.CCR-13-3141

[CR41] Hellberg V, Wallin I, Eriksson S, Hernlund E, Jerremalm E, Berndtsson M, Eksborg S, Arnér ESJ, Shoshan M, Ehrsson H (2009) Cisplatin and oxaliplatin toxicity: importance of cochlear kinetics as a determinant for ototoxicity. J Natl Cancer Inst 101:37–47. 10.1093/jnci/djn418 19116379 10.1093/jnci/djn418 PMC2639295

[CR42] Hellesnes R, Kvammen Ø, Myklebust TÅ, Bremnes RM, Karlsdottir Á, Negaard HFS, Tandstad T, Wilsgaard T, Fosså SD, Haugnes HS (2020) Continuing increased risk of second cancer in long‐term testicular cancer survivors after treatment in the cisplatin era. Int J Cancer 147:21–32. 10.1002/ijc.32704 31597192 10.1002/ijc.32704

[CR43] Herr CEW, Jankofsky M, Angerer J, Küster W, Stilianakis NI, Gieler U, Eikmann T (2003) Influences on human internal exposure to environmental platinum. J Expo Sci Environ Epidemiol 13:24–30. 10.1038/sj.jea.7500251 10.1038/sj.jea.7500251 12595881

[CR44] Herrmann J (2020) Cardiovascular toxicity with cisplatin in patients with testicular cancer: looking for something heavier than heavy metal. American College of Cardiology Foundation, Washington DC, pp 456–459. 10.1016/j.jaccao.2020.07.007 10.1016/j.jaccao.2020.07.007 PMC8352319 34396252

[CR45] Hjelle LV, Gundersen POM, Oldenburg J, Brydøy M, Tandstad T, Wilsgaard T, Fosså SD, Bremnes RM, Haugnes HS (2015) Long-term platinum retention after platinum-based chemotherapy in testicular cancer survivors: a 20-year follow-up study. Anticancer Res 35:1619–1625 25750319

[CR46] Huang KM, Leblanc AF, Uddin ME, Kim JY, Chen M, Eisenmann ED, Gibson AA, Li Y, Hong KW, DiGiacomo D (2020) Neuronal uptake transporters contribute to oxaliplatin neurotoxicity in mice. J Clin Invest 130:4601–4606. 10.1172/JCI136796 32484793 10.1172/JCI136796 PMC7456253

[CR47] Huot JR, Baumfalk D, Resendiz A, Bonetto A, Smuder AJ, Penna F (2023) Targeting mitochondria and oxidative stress in cancer-and chemotherapy-induced muscle wasting. Antioxid Redox Signal 38:352–370. 10.1089/ars.2022.0149 36310444 10.1089/ars.2022.0149 PMC10081727

[CR48] Ivanova S (2022) Comparative assessment of clinical trials, indications, pharmacokinetic parameters and side effects of approved platinum drugs. Pharmacia 69:1–7. 10.3897/pharmacia.69.e78813

[CR49] Jager A, Sleijfer S, Van Der Rijt CCD (2008) The pathogenesis of cancer related fatigue: could increased activity of pro-inflammatory cytokines be the common denominator? Eur J Cancer 44:175–181. 10.1016/j.ejca.2007.11.023 18162394 10.1016/j.ejca.2007.11.023

[CR50] Jaishankar M, Tseten T, Anbalagan N, Mathew BB, Beeregowda KN (2014) Toxicity, mechanism and health effects of some heavy metals. Interdiscip Toxicol 7:60. 10.2478/intox-2014-0009 26109881 10.2478/intox-2014-0009 PMC4427717

[CR51] Johnstone TC, Suntharalingam K, Lippard SJ (2016) The next generation of platinum drugs: targeted Pt (II) agents, nanoparticle delivery, and Pt (IV) prodrugs. Chem Rev 116:3436–3486. 10.1021/acs.chemrev.5b00597 26865551 10.1021/acs.chemrev.5b00597 PMC4792284

[CR52] Kalayda GV, Wagner CH, Jaehde U (2012) Relevance of copper transporter 1 for cisplatin resistance in human ovarian carcinoma cells. J Inorg Biochem 116:1–10. 10.1016/j.jinorgbio.2012.07.010 23010323 10.1016/j.jinorgbio.2012.07.010

[CR53] Kim WS, Kim H, Kwon KW, Im S-H, Lee BR, Ha S-J, Shin SJ (2016) Cisplatin induces tolerogenic dendritic cells in response to TLR agonists via the abundant production of IL-10, thereby promoting Th2-and Tr1-biased T-cell immunity. Oncotarget 7:33765. 10.18632/oncotarget.9260 27172902 10.18632/oncotarget.9260 PMC5085117

[CR54] Kleih M, Böpple K, Dong M, Gaißler A, Heine S, Olayioye MA, Aulitzky WE, Essmann F (2019) Direct impact of cisplatin on mitochondria induces ROS production that dictates cell fate of ovarian cancer cells. Cell Death Dis 10:851. 10.1038/s41419-019-2081-4 31699970 10.1038/s41419-019-2081-4 PMC6838053

[CR55] Kotnala S, Tiwari S, Nayak A, Bhushan B, Chandra S, Medeiros CR, Coutinho HDM (2025) Impact of heavy metal toxicity on the human health and environment. Sci Total Environ 987:179785. 10.1016/j.scitotenv.2025.179785 40466229 10.1016/j.scitotenv.2025.179785

[CR56] Langer T, am Zehnhoff-Dinnesen A, Radtke S, Meitert J, Zolk O (2013) Understanding platinum-induced ototoxicity. Trends Pharmacol Sci 34:458–469. 10.1016/j.tips.2013.05.006 23769626 10.1016/j.tips.2013.05.006

[CR57] Lazić A, Popović J, Paunesku T, Woloschak GE, Stevanović M (2020) Insights into platinum-induced peripheral neuropathy–current perspective. Neural Regen Res 15:1623–1630. 10.4103/1673-5374.276321 32209761 10.4103/1673-5374.276321 PMC7437596

[CR58] Lee J-W, Choi H, Hwang U-K, Kang J-C, Kang YJ, Kim KI, Kim J-H (2019) Toxic effects of lead exposure on bioaccumulation, oxidative stress, neurotoxicity, and immune responses in fish: a review. Environ Toxicol Pharmacol 68:101–108. 10.1016/j.etap.2019.03.010 30884452 10.1016/j.etap.2019.03.010

[CR59] Lesterhuis WJ, Punt CJA, Hato SV, Eleveld-Trancikova D, Jansen BJH, Nierkens S, Schreibelt G, de Boer A, Van Herpen CML, Kaanders JH (2011) Platinum-based drugs disrupt STAT6-mediated suppression of immune responses against cancer in humans and mice. J Clin Invest 121:3100–3108. 10.1172/JCI43656 21765211 10.1172/JCI43656 PMC3148725

[CR60] Li S, Livingston MJ, Ma Z, Hu X, Wen L, Ding H-F, Zhou D, Dong Z (2023) Tubular cell senescence promotes maladaptive kidney repair and chronic kidney disease after cisplatin nephrotoxicity. JCI Insight 8:e166643. 10.1172/jci.insight.166643 36917180 10.1172/jci.insight.166643 PMC10243740

[CR61] Li X, Jing G, Guo Z, Guo Z (2025) High-mobility group box 1 in acute kidney injury. Front Pharmacol 16:1618971. 10.3389/fphar.2025.1618971 40727103 10.3389/fphar.2025.1618971 PMC12301769

[CR62] Lin Y, Chen K, Zhao L, Zhao M, Liu Y, Li Y (2025) Dexmedetomidine attenuates oxaliplatin-induced neuropathic pain by modulating the TLR4/NF-κB pathway to reduce spinal inflammation and oxidative stress. Archiv Biochem Biophys 110572. 10.1016/j.abb.2025.110572 10.1016/j.abb.2025.110572 40744255

[CR63] Linares J, Sallent-Aragay A, Badia-Ramentol J, Recort-Bascuas A, Méndez A, Manero-Rupérez N, Lo Re D, Rivas EI, Guiu M, Zwick M (2023) Long-term platinum-based drug accumulation in cancer-associated fibroblasts promotes colorectal cancer progression and resistance to therapy. Nat Commun 14:746. 10.1038/s41467-023-36334-1 36765091 10.1038/s41467-023-36334-1 PMC9918738

[CR64] Lutsenko S, Roy S, Tsvetkov P (2025) Mammalian copper homeostasis: physiological roles and molecular mechanisms. Physiol Rev 105:441–491. 10.1152/physrev.00011.2024 39172219 10.1152/physrev.00011.2024 PMC11918410

[CR65] Makrilia N, Syrigou E, Kaklamanos I, Manolopoulos L, Saif MW (2010) Hypersensitivity reactions associated with platinum antineoplastic agents: a systematic review. Metal Based Drugs 2010:207084. 10.1155/2010/207084 20886011 10.1155/2010/207084 PMC2945654

[CR66] Maly K, Schirmer M (2015) The story of CD4+ CD28− T cells revisited: solved or still ongoing? J Immunol Res 2015:348746. 10.1155/2015/348746 25834833 10.1155/2015/348746 PMC4365319

[CR67] Manohar S, Leung N (2018) Cisplatin nephrotoxicity: a review of the literature. J Nephrol 31:15–25. 10.1007/s40620-017-0392-z 28382507 10.1007/s40620-017-0392-z

[CR68] Marullo R, Werner E, Degtyareva N, Moore B, Altavilla G, Ramalingam SS, Doetsch PW (2013) Cisplatin induces a mitochondrial-ROS response that contributes to cytotoxicity depending on mitochondrial redox status and bioenergetic functions. PLoS ONE 8:e81162. 10.1371/journal.pone.0081162 24260552 10.1371/journal.pone.0081162 PMC3834214

[CR69] McKeage MJ (1995) Comparative adverse effect profiles of platinum drugs. Drug Saf 13:228–244. 10.2165/00002018-199513040-00003 8573296 10.2165/00002018-199513040-00003

[CR70] McWhinney SR, Goldberg RM, McLeod HL (2009) Platinum neurotoxicity pharmacogenetics. Mol Cancer Ther 8:10–16. 10.1158/1535-7163.MCT-08-0840 19139108 10.1158/1535-7163.MCT-08-0840 PMC2651829

[CR71] Messori L, Merlino A (2016) Cisplatin binding to proteins: a structural perspective. Coord Chem Rev 315:67–89. 10.1016/j.ccr.2016.01.010

[CR72] Miyazawa M, Bogdan AR, Tsuji Y (2019) Perturbation of iron metabolism by cisplatin through inhibition of iron regulatory protein 2. Cell Chem Biol 26:85-97. e4. 10.1016/j.chembiol.2018.10.009 30449675 10.1016/j.chembiol.2018.10.009 PMC6338505

[CR73] Modak S, Singh H, Singh RP, Mukhopadhyay A (2017) Platinum based chemotherapy induced fatigue among cancer patients. Ann Oncol 28:x177–x178. 10.1093/annonc/mdx668.003

[CR74] Moldovan M, Rauch S, Gómez M, Palacios MA, Morrison GM (2001) Bioaccumulation of palladium, platinum and rhodium from urban particulates and sediments by the freshwater isopod *Asellus aquaticus*. Water Res 35:4175–4183. 10.1016/S0043-1354(01)00136-1 11791847 10.1016/s0043-1354(01)00136-1

[CR75] Nakao Y, Mori M, Sekiguchi Y, Morita I, Shindoh R, Mandai S, Fujiki T, Kikuchi H, Ando F, Susa K, Mori T, Waseda Y, Yoshida S, Fujii Y, Sohara E, Uchida S, Mori Y (2024) Recapitulation of Cellular Senescence, Inflammation, and Fibrosis in Human Kidney-Derived Tubuloids by Repeated Cisplatin Treatment: A Novel Pathophysiological Model for Sensing Nephrotoxicity and Drug Screening. 10.1101/2024.03.17.24304404

[CR76] Nakashima K, Aoshima M, Ohfuji S, Suzuki K, Katsurada M, Katsurada N, Misawa M, Otsuka Y, Kondo K, Hirota Y (2017) Immunogenicity of trivalent influenza vaccine in patients with lung cancer undergoing anticancer chemotherapy. Hum Vaccin Immunother 13:543–550. 10.1080/21645515.2016.1246094 27820665 10.1080/21645515.2016.1246094 PMC5360112

[CR77] O’Dwyer PJ, Stevenson JP, Johnson SW (2000) Clinical pharmacokinetics and administration of established platinum drugs. Drugs 59:19–27. 10.2165/00003495-200059004-00003 10864227 10.2165/00003495-200059004-00003

[CR78] Pabla N, Murphy RF, Liu K, Dong Z (2009) The copper transporter Ctr1 contributes to cisplatin uptake by renal tubular cells during cisplatin nephrotoxicity. Am J Physiol Renal Physiol 296:F505–F511. 10.1152/ajprenal.90545.2008 19144690 10.1152/ajprenal.90545.2008 PMC2660190

[CR79] Pachman DR, Qin R, Seisler DK, Smith EML, Beutler AS, Ta LE, Lafky JM, Wagner-Johnston ND, Ruddy KJ, Dakhil S (2015) Clinical course of oxaliplatin-induced neuropathy: results from the randomized phase III trial N08CB (Alliance). J Clin Oncol 33:3416–3422. 10.1200/JCO.2014.58.8533 26282635 10.1200/JCO.2014.58.8533 PMC4606060

[CR80] Peng J, Tao G, Chen X, Ai Y, Lai R, Liu W, Hu B, Xu Y, Xie B, Li L (2025) Cisplatin transported by COX17 induces cochlear damage by binding Myosin IIA to regulate cell pyroptosis induced by mitochondrial dysfunction. Life Sci 123961. 10.1016/j.lfs.2025.123961 10.1016/j.lfs.2025.123961 40945651

[CR81] Pignata S, De Placido S, Biamonte R, Scambia G, Di Vagno G, Colucci G, Febbraro A, Marinaccio M, Lombardi AV, Manzione L (2006) Residual neurotoxicity in ovarian cancer patients in clinical remission after first-line chemotherapy with carboplatin and paclitaxel: the multicenter italian trial in ovarian cancer (MITO-4) retrospective study. BMC Cancer 6:5. 10.1186/1471-2407-6-5 16398939 10.1186/1471-2407-6-5 PMC1361775

[CR82] Quintanilha JCF, Saavedra KF, Visacri MB, Moriel P, Salazar LA (2019) Role of epigenetic mechanisms in cisplatin-induced toxicity. Crit Rev Oncol Hematol 137:131–142. 10.1016/j.critrevonc.2019.03.004 31014509 10.1016/j.critrevonc.2019.03.004

[CR83] Rauch S, Morrison G (2000) Routes for bioaccunmulation and transormation of platinum in the urban environment. In: Zereini F, Alt F (eds) Anthropogenic Platinum-Group Element Emissions. Springer, Berlin, Heidelberg. 10.1007/978-3-642-59678-0_9

[CR84] Ravindra K, Bencs L, Van Grieken R (2004) Platinum group elements in the environment and their health risk. Sci Total Environ 318:1–43. 10.1016/S0048-9697(03)00372-3 14654273 10.1016/S0048-9697(03)00372-3

[CR85] Rébé C, Demontoux L, Pilot T, Ghiringhelli F (2019) Platinum derivatives effects on anticancer immune response. Biomolecules 10:13. 10.3390/biom10010013 31861811 10.3390/biom10010013 PMC7022223

[CR86] Reis AS, Paltian JJ, Domingues WB, Novo DL, Bolea-Fernandez E, Van Acker T, Campos VF, Luchese C, Vanhaecke F, Mesko MF, Wilhelm EA (2025) Platinum deposition in the central nervous system: a novel insight into oxaliplatin-induced peripheral neuropathy in young and old mice. Mole Neurobiol 62(3):3712–3729. 10.1007/s12035-024-04430-y 10.1007/s12035-024-04430-y 39320565

[CR87] Reith F, Campbell SG, Ball AS, Pring A, Southam G (2014) Platinum in Earth surface environments. Earth-Sci Rev 131:1–21. 10.1016/j.earscirev.2014.01.003

[CR88] Rowley B, Monestier M (2005) Mechanisms of heavy metal-induced autoimmunity. Mol Immunol 42:833–838. 10.1016/j.molimm.2004.07.050 15829271 10.1016/j.molimm.2004.07.050

[CR89] Saavedra D, García B, Lorenzo-Luaces P, González A, Popa X, Fuentes KP, Mazorra Z, Crombet T, Neninger E, Lage A (2016) Biomarkers related to immunosenescence: relationships with therapy and survival in lung cancer patients. Cancer Immunol Immunother 65:37–45. 10.1007/s00262-015-1773-6 26589409 10.1007/s00262-015-1773-6 PMC11028799

[CR90] Schierl R, Fries H-G, van de Weyer C, Fruhmann G (1998) Urinary excretion of platinum from platinum industry workers. Occup Environ Med 55:138–140. 10.1136/oem.55.2.138 9614400 10.1136/oem.55.2.138 PMC1757549

[CR91] Schofield J, Harcus M, Pizer B, Jorgensen A, McWilliam S (2024) Long-term Cisplatin nephrotoxicity after childhood cancer: a systematic review and meta-analysis. Pediatr Nephrol 39:699–710. 10.1007/s00467-023-06149-9 37726572 10.1007/s00467-023-06149-9 PMC10817831

[CR92] Schuette W, Nagel S, von Weikersthal LF, Pabst S, Schumann C, Deuss B, Salm T, Roscher K, Dickgreber N (2011) Randomized phase III trial of docetaxel plus carboplatin with or without levofloxacin prophylaxis in elderly patients with advanced non-small cell lung cancer: the APRONTA trial. J Thorac Oncol 6:2090–2096. 10.1097/JTO.0b013e3182307e3c 22052225 10.1097/JTO.0b013e3182307e3c

[CR93] Skalleberg J, Solheim O, Fosså SD, Småstuen MC, Osnes T, Gundersen POM, Bunne M (2017) Long-term ototoxicity in women after cisplatin treatment for ovarian germ cell cancer. Gynecol Oncol 145:148–153. 10.1016/j.ygyno.2017.02.006 28202195 10.1016/j.ygyno.2017.02.006

[CR94] Sliesoraitis S, Chikhale PJ (2005) Carboplatin hypersensitivity. Int J Gynecol Cancer 15:13–18. 10.1136/ijgc-00009577-200501000-00003 15670291 10.1111/j.1048-891x.2005.14401.x

[CR95] Solheim O, Tropé CG, Rokkones E, Kærn J, Paulsen T, Salvesen HB, Hagen B, Vereide AB, Fosså SD (2015) Fertility and gonadal function after adjuvant therapy in women diagnosed with a malignant ovarian germ cell tumor (MOGCT) during the “Cisplatin era.” Gynecol Oncol 136:224–229. 10.1016/j.ygyno.2014.12.010 25511159 10.1016/j.ygyno.2014.12.010

[CR96] Sprauten M, Darrah TH, Peterson DR, Campbell ME, Hannigan RE, Cvancarova M, Beard C, Haugnes HS, Fosså SD, Oldenburg J (2012) Impact of long-term serum platinum concentrations on neuro-and ototoxicity in cisplatin-treated survivors of testicular cancer. J Clin Oncol 30:300–307. 10.1200/jco.2011.37.4025 22184390 10.1200/JCO.2011.37.4025 PMC3269954

[CR97] Stelwagen J, Lubberts S, Steggink LC, Steursma G, Kruyt LM, Donkerbroek JW, van Roon AM, van Gessel AI, van de Zande SC, Meijer C (2020) Vascular aging in long-term survivors of testicular cancer more than 20 years after treatment with cisplatin-based chemotherapy. Br J Cancer 123:1599–1607. 10.1038/s41416-020-01049-3 32921790 10.1038/s41416-020-01049-3 PMC7686327

[CR98] Su L, Zhang J, Gomez H, Kellum JA, Peng Z (2023) Mitochondria ROS and mitophagy in acute kidney injury. Autophagy 19:401–414. 10.1080/15548627.2022.2084862 35678504 10.1080/15548627.2022.2084862 PMC9851232

[CR99] Kamacı SC, Koçak G, Yeşilova A, Cihan Ş (2022) Evaluation of acute and chronic nephrotoxicity in patients received cisplatin-based chemotherapy: has anything changed over time? Int Urol Nephrol 54:1085–1090. 10.1007/s11255-021-02975-8 34390437 10.1007/s11255-021-02975-8

[CR100] Tan WJT, Vlajkovic SM (2023) Molecular characteristics of cisplatin-induced ototoxicity and therapeutic interventions. Int J Mol Sci 24:16545. 10.3390/ijms242216545 38003734 10.3390/ijms242216545 PMC10671929

[CR101] Tang C, Livingston MJ, Safirstein R, Dong Z (2023) Cisplatin nephrotoxicity: new insights and therapeutic implications. Nat Rev Nephrol 19:53–72. 10.1038/s41581-022-00631-7 36229672 10.1038/s41581-022-00631-7

[CR102] Tao J, Jia S, Wang M, Huang Z, Wang B, Zhang W, Wei Y, Li W, Jiang H, Du Z (2021) Systematic identification of proteins binding with Cisplatin in blood by affinity chromatography and a four-dimensional proteomic method. J Proteome Res 20:4553–4565. 10.1021/acs.jproteome.1c00535 34427088 10.1021/acs.jproteome.1c00535

[CR103] Tchounwou PB, Yedjou CG, Patlolla AK, Sutton DJ (2012) Heavy metal toxicity and the environment. Mole Clin Environ Toxicol 3:133–164. 10.1007/978-3-7643-8340-4_6 10.1007/978-3-7643-8340-4_6 PMC4144270 22945569

[CR104] Tharmalingam MD, Matilionyte G, Wallace WHB, Stukenborg J-B, Jahnukainen K, Oliver E, Goriely A, Lane S, Guo J, Cairns B (2020) Cisplatin and carboplatin result in similar gonadotoxicity in immature human testis with implications for fertility preservation in childhood cancer. BMC Med 18:374. 10.1186/s12916-020-01844-y 33272271 10.1186/s12916-020-01844-y PMC7716476

[CR105] Turnbull AV, Rivier C (1995) Regulation of the HPA axis by cytokines. Brain Behav Immun 9:253–275. 10.1006/brbi.1995.1026 8903845 10.1006/brbi.1995.1026

[CR106] Ueda S, Yamashita S, Nakajima M, Kumamoto T, Ogawa C, Liu Y-y, Yamada H, Kubo E, Hattori N, Takeshima H (2022) A quantification method of somatic mutations in normal tissues and their accumulation in pediatric patients with chemotherapy. Proc Natl Acad Sci U S A 119:e2123241119. 10.1073/pnas.2123241119 35895679 10.1073/pnas.2123241119 PMC9351471

[CR107] Vaughan GT, Florence TM (1992) Platinum in the human diet, blood, hair and excreta. Sci Total Environ 111:47–58. 10.1016/0048-9697(92)90044-s 1542781 10.1016/0048-9697(92)90044-s

[CR108] Waissbluth S, Peleva E, Daniel SJ (2017) Platinum-induced ototoxicity: a review of prevailing ototoxicity criteria. Eur Arch Otorhinolaryngol 274:1187–1196. 10.1007/s00405-016-4117-z 27245751 10.1007/s00405-016-4117-z

[CR109] Wang Z, Xu L, Wang H, Li Z, Lu L, Li X, Zhang Q (2018) Lobaplatin-based regimens outperform cisplatin for metastatic breast cancer after anthracyclines and taxanes treatment. Saudi J Biol Sci 25:909–916. 10.1016/j.sjbs.2018.01.011 30108440 10.1016/j.sjbs.2018.01.011 PMC6087814

[CR110] Wang X, Zhou Y, Wang D, Wang Y, Zhou Z, Ma X, Liu X, Dong Y (2023) Cisplatin-induced ototoxicity: from signaling network to therapeutic targets. Biomed Pharmacother 157:114045. 10.1016/j.biopha.2022.114045 36455457 10.1016/j.biopha.2022.114045

[CR111] Wei G, Gu Z, Gu J, Yu J, Huang X, Qin F, Li L, Ding R, Huo J (2021) Platinum accumulation in oxaliplatin‐induced peripheral neuropathy. J Peripher Nerv Syst 26:35–42. 10.1111/jns.12432 33462873 10.1111/jns.12432 PMC7986112

[CR112] Weiss G, Ganz T, Goodnough LT (2019) Anemia of inflammation. Blood 133:40–50. 10.1182/blood-2018-06-856500 30401705 10.1182/blood-2018-06-856500 PMC6536698

[CR113] Yang Y, Zhao B, Gao X, Sun J, Ye J, Li J, Cao P (2021) Targeting strategies for oxaliplatin-induced peripheral neuropathy: clinical syndrome, molecular basis, and drug development. J Exp Clin Cancer Res 40:331. 10.1186/s13046-021-02141-z 34686205 10.1186/s13046-021-02141-z PMC8532307

[CR114] Zhang X, Perry RJ (2024) Metabolic underpinnings of cancer-related fatigue. Am J Physiol Endocrinol Metab 326:E290–E307. 10.1152/ajpendo.00378.2023 38294698 10.1152/ajpendo.00378.2023 PMC11901342

[CR115] Zhang F, Wang Y, Wang ZQ, Wang DS, Jiang YX, Zhang DS, Wang FH, Xu RH, Li YH (2017) Efficacy and safety of cisplatin-based versus nedaplatin-based regimens for the treatment of metastatic/recurrent and advanced esophageal squamous cell carcinoma: a systematic review and meta-analysis. Dis Esophagus 30(2). 10.1111/dote.12490 10.1111/dote.12490 27868295

[CR116] Zhang Y, Zheng J, Jiang Y, Huang X, Fang L (2020) Neglected, drug-induced platinum accumulation causes immune toxicity. Front Pharmacol 11:1166. 10.3389/fphar.2020.01166 32903504 10.3389/fphar.2020.01166 PMC7438596

[CR117] Zhang M-W, Sun X, Xu Y-W, Meng W, Tang Q, Gao H, Liu L, Chen S-H (2024) Curcumin relieves oxaliplatin‐induced neuropathic pain via reducing inflammation and activating antioxidant response. Cell Biol Int 48:872–882. 10.1002/cbin.12153 38480956 10.1002/cbin.12153

[CR118] Zhang Y, Huang X, Feng S, Chen C, Guo D, Fang L (2021) Platinum accumulation and cancer-related fatigue, correlation with IL-8, TNF-α and hemocytes. Front Pharmacol 12:658792. 10.3389/fphar.2021.658792 34557089 10.3389/fphar.2021.658792 PMC8453147

[CR119] Zhao L, Cheng Q, Wang Z, Xi Z, Xu D, Liu Y (2014) Cisplatin binds to human copper chaperone Cox17: the mechanistic implication of drug delivery to mitochondria. Chem Commun 50:2667–2669. 10.1039/C3CC48847K 10.1039/c3cc48847k 24473407

[CR120] Zoroddu MA, Aaseth J, Crisponi G, Medici S, Peana M, Nurchi VM (2019) The essential metals for humans: a brief overview. J Inorg Biochem 195:120–129. 10.1016/j.jinorgbio.2019.03.013 30939379 10.1016/j.jinorgbio.2019.03.013

